# Alpha lipoic acid efficacy in burning mouth syndrome. A controlled clinical trial

**DOI:** 10.4317/medoral.20410

**Published:** 2015-06-02

**Authors:** Begoña Palacios-Sánchez, Luis-Alberto Moreno-López, Rocío Cerero-Lapiedra, Silvia Llamas-Martínez, Germán Esparza-Gómez

**Affiliations:** 1 DMD, PhD. MD, PhD. DMD, PhD. MD, PhD. Departamento de Medicina y Cirugía Bucofacial, Universidad Complutense de Madrid. Madrid. Spain; 2 DMD, PhD Unidad de Medicina y Cirugía Oral. GAP Toledo. SESCAM. Toledo. Spain

## Abstract

**Background:**

A double-blind placebo-controlled trial was conducted in order to evaluate the efficacy of alpha lipoic acid (ALA) and determine the statistical significance of the outcome variables. Burning mouth syndrome (BMS) is defined as an oral burning sensation in the absence of clinical signs which could justify the syndrome. Recent studies suggest the existence of neurological factors as a possible cause of the disease.

**Material and Methods:**

60 patients with BMS, in two groups: case group with 600 mg/day and placebo as control group; with follow up of 2 months.

**Results:**

64% of ALA patients reported some level of improvement, with a level of maintenance of 68.75% one month after treatment. 27.6% of the placebo group also demonstrated some reduction in BMS symptoms.

**Conclusions:**

Long-term evolution and the intensity of symptoms are variables that reduce the probability of improvement with ALA treatment.

**Key words:** Burning mouth syndrome, neuropathy, alpha lipoic acid.

## Introduction

Burning mouth syndrome (BMS) is defined as a burning sensation in the oral mucosa with no clinical signs that could justify the syndrome. (1). The prevalence of BMS is 1-3% in developed countries (2), and occurs more frequently in the middle-aged and the elderly population, especially women, with a 7:1 ratio. (2). BMS is considered a syndrome as it is frequently associated with two other symptoms: xerostomia and dysgeusia (2).

In 1989, Lamey and Lewis (3) clinically classified BMS into three different types: type I, symptoms are not present upon awakening but worsen during the day; type II, symptoms are continuous throughout the day; type III, symptoms are intermittent.

The precise aetiology of BMS is still unknown, yet multiple local and systemic factors have been reported (4). Local factors associated with BMS include: hypo salivation and/or xerostomia (10-66% of cases) (5-7), parafunctional habits (7), contact allergies (8), poorly fitting prostheses (9), Candida albicans infection (10), as well as smoking, alcohol, caffeine, and very hot or spicy foods. Systemic factors associated with BMS include: menopause (5), nutritional deficiencies (vitamin B group, iron and folic acid) (5), diabetes mellitus (especially type II) (5), hypothyroidism, as well as other systemic factors, for example, a long-term pharmacologic treatment with antihypertensive drugs (11).

Regarding psychological factors, it is unclear whether these are the cause or the result of BMS. Even so, psychological factors account for BMS symptoms in more than 50% of patients, and include chronic anxiety, depression and cancer phobia (12), among others.

Scala *et al*. (13) suggest differentiating secondary BMS, when there is a local or systemic condition, from idiopathic BMS when there are no other visible alterations.

Recent studies suggest that neurological factors may be a possible cause of BMS. (14) It has been reported that alterations of the chorda tympani nerve can lead to lingual nerve hyper function resulting in the appearance of hyperalgesia (15). Data has also shown increased levels of Nerve Growth Factor (NGF) and TRPV1 channels in patients with BMS, both involved in thermal hyperalgesia (16).

Alpha lipoic acid (ALA) is a potent antioxidant that is produced naturally in the body. It can also be found in some natural foods, such as potatoes, tomatoes and spinach. To date, ALA’s main contribution is to slow down cutaneous ageing (17). It regenerates and strengthens the effects of other biological antioxidants. ALA is an efficient chelating agent for catalyzing metals in the formation of reactive oxygen species (ROS), acting against those that have already generated (18). ALA acts as a coenzyme in the production of energy (ATP), and improves glucose metabolism. In addition, ALA seems to favour the production of nerve growth factor (NGF) and has been used in the treatment of diabetic neuropathy (17,19).

There is no established treatment for BMS given its unknown aetiology. A possible neurological cause has been recently underscored. Based on this datum and the benefits of ALA in the treatment of diabetic neuropathy, there have been attempts to demonstrate the efficacy of ALA in the management of BMS. Nonetheless, the results obtained have not been conclusive due to the complexity of the variables studied.

The main objective of this study was to evaluate the efficacy of ALA over placebo in the management of BMS; as well as to determine the statistical significance of the outcome variables.

## Material and Methods

A double-blind placebo-controlled study was conducted in 60 patients clinically diagnosed with BMS. The study took place at the Departament of Oral Medicine and Surgery, Universidad Complutense of Madrid, Spain.

Diagnosis was made during the first screening phase. Patients underwent a detailed clinical evaluation and data collection sheets were completed.

The study comprised patients over 18 years of age clinically diagnosed with BMS who reported a history of continuous oral burning pain for more than 4 months with no clinical signs that could justify the syndrome (13). Patients agreed to participate in the study and signed the written consent. Exclusion criteria included: patients whose burning sensation could be related to local alterations; patients with analytical alterations and uncontrolled systemic diseases; and patients treated with cisplatin, cyclophosphamide, gentamicin and amikacin due to the possible interaction of ALA with this medication. Patients undergoing any type of BMS treatment were also excluded from the study.

After validating the inclusion criteria and obtaining the signed informed consent, patients were randomly allocated to one of the two different sequence groups: A (placebo) or B (product). The study product (group B) is ALA, Thioderm R capsules, (SesDerma, S.L. Rafelbunyol, Valencia, Spain). Placebo (group A) was a similar-looking product based on cellulose starch. This protocol was approved by the Ethical Committee of the Hospital Clínico San Carlos of Madrid.

All patients were assessed for salivary flow rates, at rest and stimulated, complete blood count and biochemistry values, including ferritin, vitamin B12 and folic acid levels.

Treatment consisted of a dose of 600 mg/day of alpha lipoic acid administered in 3 capsules of 200 mg every 8 hours for 2 months. All patients were assessed every 15 days for changes in symptomatology using a visual analog scale (VAS), as well as for the occurrence of side effects. According to VAS patients were grouped in: mild [0-3,4], moderate [3,5-6,4] and severe [6,5-10]. Changes in VAS results were achieved as: mild improvement with reduction in 50-75%; great improvement with more than 75% reduction and curation when VAS was zero. When VAS result raised it was achieved as worsened. Patients were reassessed one month after treatment.

Statistically significant differences between both groups were analysed using contingency tables and Pearson’s Chi-square test. In order to describe how the different variables may influence the results of the treatment, the logistic regression analysis was conducted independently for each of the groups.

## Results

60 Patients diagnosed with BMS were included in the study: 55 women (91.7%) and only 5 men (8.3%) with a median age of 62.13 years (range 36-86). We didn´t find any alteration in any serological variables analyzed.

The evolution time of symptomatology varied between 4 months and 20 years. The mean intensity of the symptoms evaluated by VAS (graduated from 0 to 10) was 6.6 (range 2.5-10). 38 patients (63.3%) reported a burning sensation as the most common symptom, followed by stinging (12 patients, 20%). The rest reported itching and other symptoms. The tongue was the most affected site, yet 38 patients (63.3%) reported more than one site. According to Lamey and Lewis’s BMS classification, 38 patients (63.3%) belonged to type I, 17 patients (28.3%) to type II, and only 5 patients (8.3%) to type III.

In addition to burning sensation, 10 patients (16.7%) reported dysgeusia, and 13 (21.7%) xerostomia. 24 patients (40%) reported having both symptoms at the same time. A reduction in both stimulated and unstimulated salivary flow was found in 25 patients (41.7%).

In our study, 20 patients (33.3%) associated the onset of their BMS symptoms with a dental treatment. 16 patients (26.7%) related BMS with personal and/or family issues.

Regarding BMS and its association with systemic pathologies, 19 patients (31.7%) showed none, while 13 (21.7%) had high blood pressure, and 8 (13.3%) hypothyroidism. 19 patients (31.7%) presented other pathologies as diabetes mellitus, osteoporosis, arthrosis and gastrointestinal alterations.

To assess level of depression we used the Beck Depression Inventory (BDI). BSI is the most widely used instrument for detecting depression the BDI–II consists of 21 items to assess the intensity of depression in clinical and normal patients. Each item is a list of four statements arranged in increasing severity about a particular symptom of depression.

59 patients filled out the questionnaire. 32 (54.2%) patients showed some level of depression.

Regarding the use of medication, in our study 53.3% of patients used some type of psycho tropic drug (antidepressants and/or anxiolytics), 25% antihypertensives (11.7% ACE inhibitors, 8.3% beta-blockers, 3.3% diuretics and 1.7% ACE inhibitor +beta-blocker), 11.7% levothyroxine and 43.3% other medication (antacids, analgesics and anti diabetics).

60 patients agreed to participate in the study, but only 54 completed the trial: 29 patients belonged to group A (placebo) and 25 to group B (ALA). Patients were reassessed every 15 days after the beginning of the treatment. Final results were obtained after two months according to the level of variation on VAS scored by the patient and the self-reported description.

Since these are qualitative variables, results were analysed using contingency tables and Pearson’s Chi-square test. Results were divided into three categories: improvement (slight improvement, decided improvement and resolution), no change and worse.

8 of the 29 patients treated with placebo showed some level of improvement (27.5%), 5 worsened (17.2%) and 16 experimented no change in symptomatology (55.2%). 16 (64%) of the 25 patients treated with ALA improved, 9 (36%) showed no changes, and none worsened. Statistically significant differences between both groups were established when *p*<0.05 (*p*=0,009) was obtained from the Chi-square test. Comparing the ALA and placebo groups, it should be noted that none of the ALA patients reported worsening during the trial, and the possible placebo effect in BMS, given that 30% of the patients treated with placebo improved at the end of the treatment. The results are summarized in  Table 1.

**Table 1:**
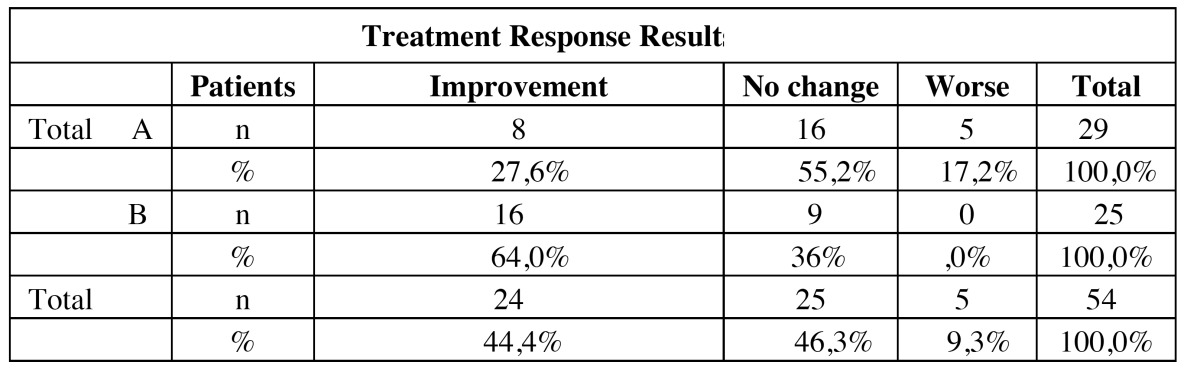
Results of treatment.

Patients were reassessed one month after treatment. 4 of the 8 patients who had improved during treatment with placebo (Group A) reported a relapse of burning. 5 (31.25%) of the 16 patients with signs of improvement during ALA treatment (Group B) worsened one month after treatment was concluded.

**Figure 1:**
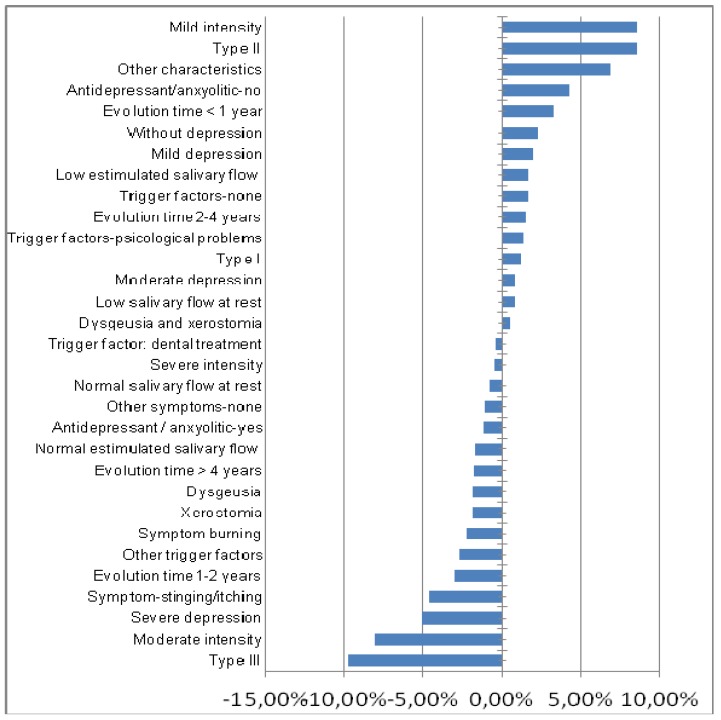
Influence of Variables in treatment response to ALA (% ). (Negative values indicate worse response to ALA).

In order to describe how the different variables may influence the results of the treatment, the logistic regression analysis was conducted independently for each of the groups. The placebo group did not have statistical significance, yet the ALA group showed significant results. Consequently, patients without depression or with mild-moderate depression, experiencing low symptom intensity and an evolution time of less than a year are more likely to improve. However, patients undergoing antidepressant and/or anxiolytic therapy, who had severe symptoms and associated their onset with some dental procedure, dysgeusia and xerostomia as well as burning, and an evolution time of more than 4 years, showed less likelihood of improving with ALA (Fig. 1).

## Discussion

There are no definitive therapies for BMS (20). According to published data, ALA is reportedly one of the most effective drugs in the management of BMS. However, to date, there is no clear consensus (21).

Femiano *et al*. have published most of the studies that examine the efficacy of ALA in the management of BMS (22-26). They use a dose of 600 mgr/day with a follow up around 30 days in their studies, as our study. Their results have been quite effective for ALA in order to improve symptoms, very similar in general compared with our results. There are some differences to consider that we point below.

In their first study (22), they carried out an open trial of 42 patients to compare ALA treatment (600 mg/day) over placebo. In the study group, none of the patients worsened, similar to our study. 24% presented no changes and 76% reported some level of improvement. However, statistical analysis was not conducted, the sample size was small, and treatment duration was only one month.

Femiano and Scully (23) evaluated ALA at a dose of 600 mg/day in a 60-patient double-blind placebo controlled study. 97% of the patients reported some improvement with ALA, 3% showed no changes in symptomatology, and none of the patients reported worsening of symptoms. These results were statistically significant in favour of ALA. Similarly to our study, not a single patient treated with ALA worsened; but the results obtained with ALA (97% improvement) were higher than our own results. We must also highlight the important placebo effect obtained (40%).

In 2002, Femiano *et al*. (24) compared ALA with other products (Bethanechol 5mg/8 hours, Lactoperoxidase and 3% Xylitol). Administration of ALA showed statistically significant results.

Femiano *et al*. (25) in 2003 conducted an open trial of 192 patients divided into four groups: one undergoing cognitive therapy (CT), one treated with ALA (600mg/day), one combining CT and ALA, and one with placebo. The ALA group and the ALA + CT group showed statistically significant improvement compared to CT alone and the placebo group.

In 2004, Femiano *et al*. (26) studied the efficacy of ALA in patients treated with and without anxiolytics. Although the latter group displayed more significant results, these were not statistically significant. In 2011, López-D’Alessandro and Escovich verified the efficacy of ALA (600 mg/day) in conjunction with gamma-amino butyric acid (GABA) over placebo over a two-month period. The combination ALA + GABA proved to be the most effective according to patients’ perceptions (27).

Other authors have used ALA in higher concentrations. In 2008, Carbone *et al*. (28) conducted a 60-patient double-blind study to evaluate the efficacy of pure ALA 800 mg/day over ALA 800 mg/day supplemented with a vitamin complex, and in comparison with placebo. The 3 groups showed a reduction in symptoms, but with no statistical significance. López-Jornet *et al*. (29) used the same concentration of ALA over placebo and equally found that both groups improved, yet with no statistically significant differences.

Finally, Cavalcanti *et al*. (30), in their crossover trial comparing the efficacy of ALA (600mg/day) over placebo, found a reduction in symptoms in both groups. The rate of this reduction was higher in the first month than in the second month of treatment.

ALA appears to have benefits in the management of BMS, yet the results supporting its efficacy is inconclusive. Further studies, with a higher number of patients, are, therefore, needed.

Variables such as depression, long-term evolution and symptom intensity make it less likely for ALA patients to improve. This fact, together with the importance of the placebo effect, suggests the need to assess the psychological and/or psychiatric implications that may require multidisciplinary treatments.
